# Prevalence and associated factors of workplace violence against nurses in different-level hospitals: a cross-sectional study in Lanzhou, China

**DOI:** 10.3389/fpubh.2025.1737881

**Published:** 2025-12-10

**Authors:** Fei Wang, Jinyu Wang, Yuting Li, Wenjie Liu, Sheng Li

**Affiliations:** 1 School of Public Health, Gansu University of Chinese Medicine, Lanzhou, China; 2 School of Public Health, Lanzhou University, Lanzhou, China; 3 First Clinical Medical College, Gansu University of Chinese Medicine, Lanzhou, China; 4 The No. 2 People's Hospital of Lanzhou, Lanzhou, China

**Keywords:** workplace violence, nursing staff, influencing factors, occupational wellbeing, cross-sectional study

## Abstract

**Background:**

Workplace violence (WPV) is a severe challenge faced by healthcare systems globally, posing significant threats to the occupational safety and wellbeing of nursing staff. This study aims to understand the prevalence of WPV experienced by nursing personnel in different levels of hospitals in Lanzhou, China, analyze its key influencing factors, and explore the negative impact of WPV on the occupational wellbeing of nurses.

**Methods:**

This study employed a cross-sectional survey design, conducted from February to March 2025, utilizing a multi-stage sampling method to survey nursing staff from five tertiary hospitals and five secondary hospitals in Lanzhou. A total of 1,702 valid questionnaires were collected. Data were gathered using the “Questionnaire on Workplace Violence Exposure Among Healthcare Workers” and the “Occupational Wellbeing Scale for Healthcare Workers.” Descriptive analysis, chi-square tests, Wilcoxon rank-sum tests, and multivariate logistic regression analysis were performed using SPSS 27.0.

**Results:**

The study found that the overall incidence rate of WPV among nursing staff in the past year was 69.92%, with verbal abuse being the most common form (69.04%). The incidence rate of WPV in tertiary hospitals (74.97%) was significantly higher than that in secondary hospitals (64.86%; *P* < 0.001). Multivariate logistic regression analysis indicated that hospital level, affiliation, department, education level, parental status, and years of service were independent influencing factors of WPV (*P* < 0.05). Nurses who experienced WPV scored significantly lower in all dimensions of occupational wellbeing (physical and mental health, value realization, social support, financial income, and work environment) compared to those who did not experience WPV (*P* < 0.001).

**Conclusion:**

The incidence rate of WPV among nursing staff in Lanzhou is at a high level and is closely related to hospital tier, department type, and individual characteristics. WPV significantly reduces the occupational wellbeing of nurses. The findings suggest that healthcare institutions should implement tiered and targeted intervention strategies, enhancing organizational support, optimizing violence prevention measures, and providing psychological interventions to effectively improve the occupational safety and wellbeing of nursing staff.

## 1 Introduction

Workplace violence (WPV) refers to acts or threats of physical assault, verbal abuse, intimidation, sexual harassment, or property damage that endanger the safety, health, or wellbeing of workers within occupational environments (1, 2). Nurses, as a high-risk occupational group, face elevated exposure to WPV due to frequent interactions with patients and their families in high-stress clinical settings (3). Globally, ~2 million nursing professionals experience WPV annually (4), with prevalence rates in China ranging between 77.6 and 78.0% (5, 6), resulting in long-term physical, psychological, and professional consequences. WPV represents one of the most complex and hazardous occupational threats in healthcare. A synthesis of 68 studies highlights its severe repercussions on healthcare workers' physical health (e.g., injuries), psychological wellbeing (e.g., anxiety, PTSD), and job performance (e.g., reduced productivity) (7). Beyond individual harm, WPV generates substantial economic burdens through lost workdays, diminished workforce capacity, and costs associated with safety infrastructure upgrades (8, 9). It also correlates with increased turnover intentions among healthcare professionals (10, 11), elevated organizational costs, and compromised care quality (12). Chronic exposure to WPV exacerbates occupational stress and burnout risks (10), while eroding trust in societal norms and professional values. Despite its pervasive impact, critical gaps persist in evaluating the efficacy of existing hospital-based interventions (13). The frequent occurrence of WPV against nurses worldwide underscores the urgency of understanding its epidemiology and modifiable determinants. Comprehensive analysis of WPV incidence patterns and context-specific risk factors is essential for designing tailored interventions to mitigate this escalating public health challenge.

## 2 Study participants and methods

### 2.1 Study population

This cross-sectional study was conducted in Lanzhou City, China, between February and March 2025. To enhance sample representativeness, a multi-stage sampling method was employed: hospitals were first stratified by level (tertiary vs. secondary), followed by random selection of 5 tertiary hospitals and five secondary hospitals within Lanzhou. Questionnaires were distributed to all eligible nurses in the selected hospitals with coordination from nursing departments. To ensure adequate statistical power for comparative analysis across hospital tiers, this study employed stratified sampling and aimed to secure comparable-sized samples in both tertiary and secondary hospitals. Through coordination with the nursing departments of participating institutions, 851 valid questionnaires were ultimately collected from each tier. We acknowledge that this non-proportional, equally sized sampling approach may not fully represent the precise demographic distribution of nurses across hospital tiers in Lanzhou, a limitation that will be further addressed in Section 4. Inclusion criteria were: ① ≥1 year of clinical experience, ② possession of a valid nursing practice certificate, and ③ voluntary participation with signed informed consent. Exclusion criteria included: ① visiting nurses, ② nurses undergoing standardized training, and ③ student interns. All participants in this study provided electronic informed consent forms, with no minors involved in the research. This study was conducted in accordance with the Declaration of Helsinki and was approved by the Ethics Committee of the First People's Hospital of Lanzhou (Approval No.: 2025A-8).

### 2.2 Research methods

#### 2.2.1 Demographic survey

A self-administered demographic questionnaire was developed to collect data across 14 sociodemographic dimensions. The survey items included: gender, age, educational attainment, professional title, monthly income, administrative role, years of service, institutional affiliation (hospital ownership), department, hospital level, employment type, marital status, number of children, and leisure interests.

#### 2.2.2 Healthcare workers' workplace violence investigation

The Questionnaire on Workplace Violence Exposure Among Healthcare Workers, developed by Chen et al. (14) at Southern Medical University, was utilized in this study. Specifically, the first part of the questionnaire was employed to investigate the types and frequency of workplace violence experienced by healthcare workers within the past 12 months, encompassing three categories: verbal violence, physical violence, and sexual harassment. Each category adopted a 4-tier scoring system: responses of “0 times,” “1 time,” “2–3 times,” and “>3 times” were assigned 0, 1, 2, and 3 points, respectively. The total score ranged from 0 to 9 points, with higher scores indicating greater exposure frequency (0 points: no exposure; 1–3 points: low frequency; 4–6 points: moderate frequency; 7–9 points: high frequency). The questionnaire exhibited moderate internal consistency in this study, with a Cronbach's α coefficient of 0.633. Though the Cronbach's alpha coefficient for this section demonstrated moderate reliability in our study, we retained the scale given its proven utility through extensive application in domestic WPV research involving healthcare professionals, as well as its structural conciseness and adaptability for large-scale epidemiological investigations.

#### 2.2.3 Occupational wellbeing assessment

The Occupational Wellbeing Scale for Healthcare Workers, originally developed by Hu Dongmei (15), was adapted in this study to evaluate professional fulfillment. This 24-item instrument assesses five dimensions: physical and mental health (six items), value/competence realization (six items), social support (five items), economic satisfaction (three items), and work environment (four items). Each item employs a five-point Likert scale (1 = strongly agree, 2 = partially agree, 3 = neutral, 4 = partially disagree, 5 = strongly disagree), with higher total scores indicating greater occupational wellbeing. Notably, the six items in the physical and mental health dimension required reverse scoring prior to total score calculation. The scale demonstrated excellent reliability with a Cronbach's α coefficient of 0.869 in the current sample.

#### 2.2.4 Survey methodology

The study employed electronic questionnaires distributed via the Questionnaire Star platform. Following formal approval from participating hospitals' nursing departments, research team members coordinated with nurse managers to conduct systematic training sessions elucidating the survey objectives, assessment content, academic significance, and operational protocols. Emphasis was placed on ensuring questionnaire anonymity and data confidentiality, with explicit reinforcement of inclusion/exclusion criteria. Nurse managers distributed questionnaires to staff nurses, requiring completion under anonymized conditions after obtaining informed consent. Technical constraints were implemented to restrict participation to a single submission per device/IP address, with mandatory responses for all items. Post-collection, rigorous data cleaning was performed to exclude patterned responses and questionnaires completed in under 2 min.

#### 2.2.5 Statistical analysis

Data analysis was conducted using SPSS 27.0. Normality of continuous variables was assessed via the Shapiro-Wilk test. Due to non-normal distributions identified in both the Workplace Violence Exposure Questionnaire and the Occupational Wellbeing Scale, continuous variables were expressed as median and interquartile range [M (P25, P75)], with group comparisons performed using the Wilcoxon rank-sum test. Categorical variables were presented as frequency (percentage), and intergroup differences were analyzed by chi-square test. Statistically significant variables identified through univariate analysis were incorporated into a multivariate logistic regression model, with workplace violence exposure status as the dependent variable, to explore independent risk factors. Variance Inflation Factors (VIF) ranged from 1.015–1.552, well below the threshold of 5, indicating no significant multicollinearity. The significance level was set at α = 0.05.

## 3 Results

### 3.1 Comparison of workplace violence prevalence among nursing staff across hospital tiers

A total of 1,800 questionnaires were distributed, with 1,702 valid responses collected (valid response rate: 94.56%), including 851 participants from tertiary hospitals and 851 from secondary hospitals. The study identified 1,190 nurses (69.92%) exposed to workplace violence within the past year. Comparative analysis revealed significant disparities in exposure rates between hospital tiers: tertiary hospitals demonstrated markedly higher prevalence (74.97%, *n* = 638) compared to secondary hospitals (64.86%, *n* = 552). Violence type distribution showed statistically significant variations (χ^2^ = 1,351.963, *P* < 0.001), with verbal aggression being most prevalent (69.04%) and sexual harassment least frequent (14.28%; see Table 1). Frequency stratification further highlighted tier-specific patterns (χ^2^ = 32.907, *P* < 0.001): tertiary hospital nurses exhibited higher overall risk, evidenced by a lower proportion of zero-exposure cases (25.03 vs. 35.14% in secondary hospitals) and a 1.6-fold greater moderate-frequency exposure rate (21.03 vs. 12.93%; see Table 2).

**Table 1:** Frequency distribution of workplace violence types among nursing staff (*n* = 1,702).

**Types of violence**	**0 times [*n* (%)]**	**1 time [*n* (%)]**	**2–3 times [*n* (%)]**	**>3 times [*n* (%)]**	**Total incidence (≥1 time; %)**
Verbal abuse	527 (30.96)	316 (18.57)	354 (20.80)	505 (29.67)	69.04
Physical violence	1,226 (72.03)	224 (13.16)	145 (8.52)	107 (6.29)	27.97
Sexual harassment	1,459 (85.72)	140 (8.23)	83 (4.88)	20 (1.18)	14.28

**Table 2:** Frequency comparison of workplace violence exposure among nursing staff across hospital tiers.

**WPV frequency categories**	**Tertiary hospitals (*n* = 851) [*n* (%)]**	**Secondary hospitals (*n*=851) [*n* (%)]**
No exposure	213 (25.03)	299 (35.14)
Low-frequency exposure	418 (49.12)	391 (45.95)
Moderate-frequency exposure	179 (21.03)	110 (12.93)
High-frequency exposure	41 (4.82)	51 (5.99)

### 3.2 Impact of workplace violence on occupational wellbeing

**Figure 1:**
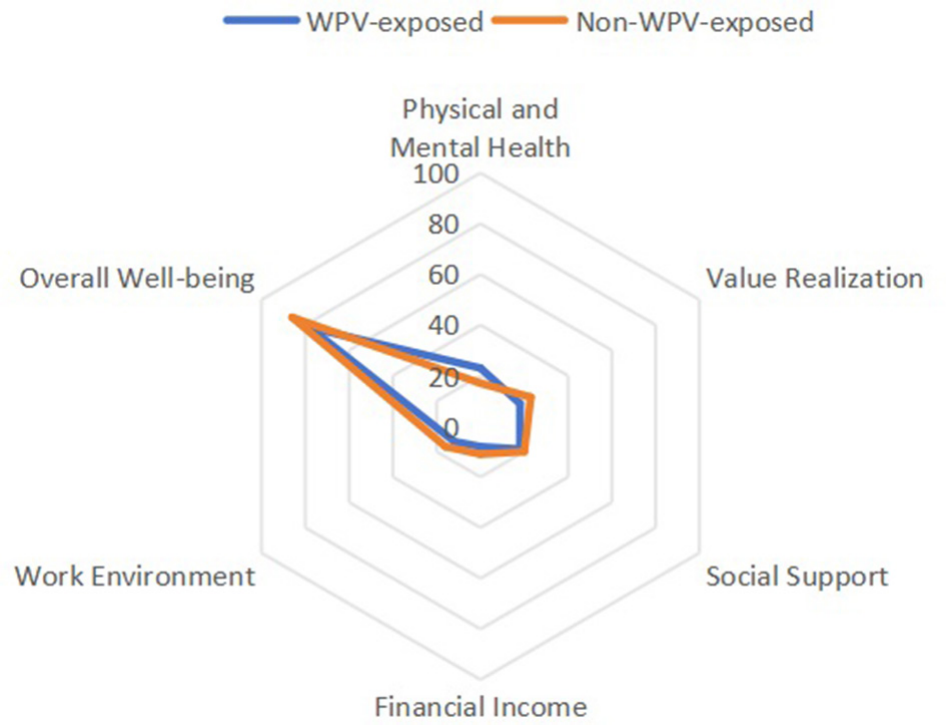
Radar chart comparing occupational wellbeing dimensions between nurses exposed and non-exposed to workplace violence. Scores for each of the five dimensions (Physical and Mental Health, Value Realization, Social Support, Financial Income, and Work Environment) were normalized to a 0–100 scale for comparative visualization. The chart illustrates that nurses in the WPV-exposed group (solid line) reported consistently lower scores across all dimensions compared to their non-exposed counterparts (dashed line), with the most pronounced disparities observed in the Work Environment and Physical and Mental Health domains.

This study revealed that nursing staff exposed to workplace violence exhibited significantly lower occupational wellbeing scores across all dimensions compared to their non-exposed counterparts (*P* < 0.001). The median overall wellbeing score for violence-exposed nurses was 78 points, markedly lower than the 86 points observed in non-exposed groups. The largest effect sizes emerged in the physical and mental health (*r* = 0.363) and work environment (*r* = 0.374) dimensions, indicating robust associations between violence exposure and wellbeing deterioration in these domains. The overall effect sizes ranged from *r* = 0.270 to 0.374, demonstrating moderate correlations between violence exposure and reduced wellbeing (see Table 3 and Figure 1). These findings compellingly demonstrate that workplace violence transcends mere safety incidents, functioning as a critical erosive factor of professional fulfillment among nursing staff.

**Table 3:** Comparison of occupational wellbeing dimensions between WPV-exposed and non-exposed nursing staff [median (P25, P75)].

**Dimensions**	**WPV-exposed (*n* = 1,190)**	**Non-WPV-exposed (*n* = 512)**	***Z***	***P*-value**	**Effect size (*r*)**
Physical and mental health	23 (19, 26)	17 (12, 22)	−14.977	< 0.001	0.363
Value realization	18 (14, 22)	23 (18, 26)	−13.109	< 0.001	0.318
Social support	18 (15, 20)	20 (18, 24)	−12.373	< 0.001	0.300
Financial income	8 (5, 10)	11 (8, 12)	−13.106	< 0.001	0.318
Work environment	12 (10, 15)	16 (13, 18)	−15.436	< 0.001	0.374
Overall wellbeing	78 (69, 86)	86 (77, 94)	−11.151	< 0.001	0.270

### 3.3 Univariate analysis of workplace violence exposure among nursing staff

The univariate analysis identified hospital grade, hospital affiliation, gender, department, administrative position, income, education level, marital status, number of children, and years of service as variables demonstrating significant associations with workplace violence exposure (*P* < 0.05), while age, professional title, job type, and personal hobbies showed no significant associations (*P* > 0.05). Subgroup analysis revealed elevated violence prevalence among the following groups: nurses in tertiary hospitals, provincial/municipal-affiliated institutions, males, emergency department staff, non-administrative clinical workers, those with a monthly income exceeding ¥6,000, bachelor's degree holders or higher, divorced/widowed individuals, staff without children or with one child, and nurses with 6–10 or >20 years of service (see Table 4).

**Table 4:** Univariate analysis of workplace violence (WPV) exposure among nursing staff [*n* (%)].

**Variable**	**Number of cases**	**WPV exposure**		**χ^2^**	***P*-value**
		**Yes (*****n*** = **1,190)**	**No (*****n*** = **512)**		
**Hospital level**				20.660	< 0.001
Tertiary	851	638 (74.97)	213 (25.03)		
Secondary	851	552 (64.86)	299 (35.14)		
**Hospital affiliation**				322.993	< 0.001
Provincial	288	266 (92.36)	22 (7.64)		
Municipal	383	369 (96.34)	14 (3.66)		
District/county or below	1,031	555 (53.83)	476 (46.17)		
**Gender**				10.127	0.001
Male	83	71 (85.54)	12 (14.46)		
Female	1,619	1,119 (69.12)	500 (30.88)		
**Age**				5.484	0.064
< 31	783	545 (69.60)	238 (30.40)		
31–40	731	500 (68.40)	231 (31.60)		
>41	188	145 (77.13)	43 (22.87)		
**Department**				29.732	< 0.001
Emergency department	155	135 (87.1)	20 (12.9)		
Clinical departments	1,407	960 (68.23)	447 (31.77)		
Paraclinical departments	120	86 (71.67)	34 (28.33)		
Administrative departments	20	9 (45.00)	11 (55.00)		
**Professional title**				1.925	0.750
Junior	1,088	755 (69.39)	333 (30.61)		
Intermediate	521	371 (71.21)	150 (28.79)		
Associate senior	57	41 (71.93)	16 (28.07)		
Full senior	1	1 (100.00)	0 (0.00)		
Other	35	22 (62.86)	13 (37.14)		
**Administrative position**				19.108	< 0.001
Department director/deputy	3	1 (33.33)	2 (66.67)		
Head nurse	152	84 (55.26)	68 (44.74)		
General medical staff	1,547	1,105 (71.43)	442 (28.57)		
**Employment type**				2.497	0.114
Formally established staff	283	209 (73.85)	74 (26.15)		
Contract-based employment	1,419	981 (69.13)	438 (30.87)		
**Monthly income (RMB)**				37.024	< 0.001
< 3,000	663	425 (64.10)	238 (35.90)		
3,000–6,000	923	659 (71.40)	264 (28.60)		
>6,000	116	106 (91.38)	10 (8.62)		
**Education level**				43.119	< 0.001
Associate degree or below	512	301 (58.79)	211 (41.21)		
Bachelor's degree or above	1,187	889 (74.71)	301 (25.36)		
**Marital status**				13.893	0.001
Unmarried	431	317 (73.55)	114 (26.45)		
Married	1,238	842 (68.01)	396 (31.99)		
Divorced/widowed	33	31 (93.94)	2 (6.06)		
**Parental status**				64.634	< 0.001
Childless	634	452 (71.29)	182 (28.71)		
One child	603	476 (78.94)	127 (21.06)		
Two or more children	465	262 (56.34)	203 (43.66)		
**Years of service**				33.482	< 0.001
≤ 5	502	319 (63.55)	183 (36.45)		
6–10	557	427 (76.66)	130 (23.34)		
11–20	492	323 (65.65)	169 (34.35)		
>20	151	121 (80.13)	30 (19.87)		
**Hobbies**				0.598	0.741
None	571	403 (70.58)	168 (29.42)		
1–2 hobbies	975	675 (69.23)	300 (30.77)		
Multiple (>3)	156	112 (71.79)	44 (28.21)		

### 3.4 Multivariate analysis of workplace violence exposure among nursing staff across hospital grades

With workplace violence exposure as the dependent variable, variables demonstrating statistical significance in univariate analyses were incorporated into a stepwise logistic regression model. Hospital grade, hospital affiliation, department, education level, parenthood status, and years of service were identified as independent risk-associated variables (*P* < 0.05). Compared to secondary hospitals, tertiary hospital nurses faced 2.290-fold higher violence risk (OR = 2.290, *P* < 0.001). Provincial hospital staff showed 15.515-fold greater risk vs. county-level institutions (OR = 15.515, *P* < 0.001), while municipal hospitals exhibited the highest risk (OR = 30.562, *P* < 0.001). Emergency department personnel had 11.626-times elevated risk compared to administrative staff (OR = 11.626, *P* < 0.001). Nurses with bachelor's degrees incurred 1.533-fold higher risk than those with associate degrees or below (OR = 1.533, *P* < 0.01). Childless nurses (OR = 2.656, *P* < 0.001) and those with one child (OR = 2.362, *P* < 0.001) demonstrated greater vulnerability than counterparts with two or more children. Regarding seniority, nurses with 6–10 years (OR = 2.474), 11–20 years (OR = 1.873), and >20 years of service (OR = 2.485) all exhibited significantly elevated risks vs. those with ≤ 5 years' experience (all *P* < 0.01; see Table 5).

**Table 5:** Multivariate logistic regression analysis of workplace violence exposure among nursing staff.

**Variable**	**Coefficient**	**SE**	**Wald χ^2^**	***P*-value**	**OR (95% CI)**
**Hospital level**					
Secondary					1.000
Tertiary	0.828	0.145	32.538	< 0.001	2.290 (1.723–3.044)
**Hospital affiliation**					
District/county or below					1.000
Provincial	2.742	0.255	115.944	< 0.001	15.515 (9.419–25.556)
Municipal	3.420	0.295	134.033	< 0.001	30.562 (17.130–54.528)
**Department**					
Administrative departments					1.000
Emergency department	2.453	0.586	17.501	< 0.001	11.626 (3.684–36.693)
Clinical departments	0.838	0.528	2.524	0.112	2.312 (0.822–6.505)
Paraclinical departments	1.044	0.578	3.268	0.071	2.841 (0.916–8.815)
**Education level**					
Associate degree or below					1.000
Bachelor's degree or above	0.427	0.138	9.550	0.002	1.533 (1.169–2.009)
**Parental status**					
Two or more children					1.000
Childless	0.977	0.205	22.753	< 0.001	2.656 (1.778–3.967)
One child	0.860	0.165	27.202	< 0.001	2.362 (1.710–3.263)
**Years of service**					
≤ 5					1.000
6–10	0.906	0.189	23.004	< 0.001	2.474 (1.709–3.583)
11–20	0.627	0.219	8.233	0.004	1.873 (1.220–2.874)
>20	0.910	0.301	9.154	0.002	2.485 (1.378–4.480)
Constant	−2.819	0.587	23.094	< 0.001	0.060

## 4 Discussion

This study revealed a substantial WPV prevalence rate of 69.92% among nursing staff in Lanzhou over the past year. This finding aligns with domestic reports focusing on high-risk departments (e.g., emergency and psychiatric units) (16, 17), yet significantly exceeds national survey outcomes (18) and the global average reported by the World Health Organization (WHO) (19). Such discrepancies may stem from methodological variations in research instruments, sampling frameworks, regional cultural contexts, and operational definitions of violent acts (20). Nevertheless, the consistently elevated rates unequivocally demonstrate that WPV remains a pervasive and urgent challenge within Chinese healthcare systems. Regarding violence typology, verbal aggression emerged as the most prevalent form, indicating that non-physical hostility constitutes the primary stressor encountered by nursing staff—a pattern corroborated by both domestic and international studies (21, 22). Notably, tertiary hospitals exhibited significantly higher WPV incidence and frequency than secondary institutions. Multivariate logistic regression further quantified this disparity, showing 2.29-fold greater violence risk for tertiary hospital nurses compared to secondary hospital counterparts. This gradient likely reflects tertiary hospitals' role as regional medical hubs managing critically ill patients with complex conditions, where heightened patient/family expectations intersect with systemic stressors (23). Concurrently, structural tensions arising from resource constraints – manifested in prolonged wait times and fragmented care pathways—collectively exacerbate clinician-user conflicts, amplifying violence risks (22).

Multivariate logistic regression analysis further delineated independent risk factors for WPV. Hospital grade, administrative affiliation, department, education level, parenthood status, and years of service were identified as key correlates of WPV occurrence. Foremost, nurses in tertiary, municipal, and provincial hospitals faced substantially higher risks—a pattern reaffirming the intensified pressures faced by high-tier medical centers. Departmental disparities proved most striking, with emergency department nurses confronting 11.626 times greater risk than administrative staff (OR = 11.626, *P* < 0.001). This aligns with the inherent volatility of emergency settings characterized by unpredictable workloads, emotionally charged patient interactions, and time-sensitive decision-making—an association well-documented in existing literature (24, 25). Individual characteristics revealed nuanced risk associations. Childless nurses and those with one child exhibited greater vulnerability than counterparts with two or more children, possibly reflecting differential family support systems, societal role expectations, and stress-coping capacities. Elevated risks among nurses with 6–10 and >20 years of service may denote career-stage-specific challenges: the former group likely navigates high clinical competency with nascent stress management skills, while veterans face cumulative effects of burnout and complex clinician-user conflicts (26). Contrary to some findings, bachelor's degree holders or higher demonstrated increased susceptibility (OR = 1.533, *P* < 0.01). This paradox may stem from their disproportionate involvement in high-stakes communication—explaining intricate diagnoses and coordinating multidisciplinary care in advanced healthcare settings—thus amplifying exposure to conflict-prone scenarios (27).

A pivotal revelation of this study concerns the profound erosion of occupational wellbeing induced by WPV. The data demonstrated significantly lower wellbeing scores among violence-exposed nurses across five dimensions: physical/mental health, professional value fulfillment, social support, financial compensation, and work environment—with the strongest inverse associations observed in physical/mental health (*r* = −0.374) and work environment (*r* = −0.363). This compellingly substantiates that WPV functions not merely as an immediate safety hazard, but operates as a key corrosive agent chronically impairing nurses‘ psychological health and degrading their professional fulfillment (28). The adverse psychological effects of WPV on nurses may operate through sustained depletion of psychological resources, leading to job burnout and emotional exhaustion (29), and demonstrate significant associations with depression, anxiety, post-traumatic stress disorder (PTSD), and turnover intention (30, 31). Such chronic psychological strain not only compromises nurses' personal wellbeing but may also indirectly jeopardize patient safety and care quality through diminished clinical performance (32, 33).

Based on research findings, we propose the following targeted interventions: (1) Tiered precision interventions: Prioritize security and training resource allocation in high-risk areas such as tertiary hospitals and emergency departments, including deploying 24-h security personnel, installing emergency alarm systems, and optimizing triage processes to reduce patient waiting times (34). Concurrently, ensure adequate nurse staffing to prevent violence escalation caused by workforce shortages and excessive workloads (35). (2) “Zero-tolerance” support systems: Hospitals should establish and strictly enforce anti-violence policies, implement secure and accessible reporting mechanisms, and ensure transparent investigation procedures. Comprehensive support for victimized nurses must include psychological counseling, legal assistance, and paid leave to mitigate secondary trauma while cultivating a positive safety culture (36–38). (3) Systematic non-technical competency training: Implement structured programs focusing on communication techniques, conflict de-escalation, and emotional regulation. High-risk departments should conduct regular scenario simulation drills to enhance violence identification and real-time response capabilities (25, 39). (4) High-risk group support and psychological capital development: Provide personalized interventions including flexible scheduling, mentorship programs, and peer networks for vulnerable nurses (childless staff, first-time mothers, specific tenure groups). Integrate psychological capital enhancement into routine training through expert-led workshops and resilience-building seminars to strengthen mental fortitude and adaptive capacities (40).

While yielding valuable insights, this study exhibits several limitations. First, the cross-sectional design precludes causal inferences between predictors and WPV occurrence—for instance, whether adverse work environments precipitate violence or vice versa. Future research could employ longitudinal designs to track WPV's temporal dynamics and long-term psychosocial consequences. Second, self-reported questionnaire data risks recall bias and social desirability distortions, potentially underestimating/hyperestimating WPV prevalence. Though anonymous surveys were administered, subsequent studies should triangulate findings with hospitals' official adverse event records for enhanced validity. The WPV assessment tool employed in this study demonstrated moderate reliability (Cronbach's α = 0.633), potentially compromising the stability of measurement outcomes. The exceptionally high odds ratio (OR) value may indicate potential risk clustering phenomena within specific healthcare environments. Third, geographic confinement to Lanzhou may constrain generalizability. Furthermore, this study did not encompass all potential explanatory variables, including nurses' shift patterns (e.g., night shifts), granular workload metrics, and unit-specific patient flow characteristics, which may constitute significant determinants of workplace violence.

Future research directions should prioritize the following areas: First, employing longitudinal or mixed-methods approaches to monitor dynamic WPV trends and their long-term impacts on nurses' professional development, complemented by qualitative interviews to explore root causes (41); Second, conducting intervention studies to systematically evaluate the cost-effectiveness of strategies such as security system upgrades, staff training modules, cultural transformation initiatives, and psychological capital enhancement programs, thereby informing evidence-based practices (34); Third, expanding the research scope by investigating both externally perpetrated WPV (e.g., by patients or families) and internally originated WPV (e.g., by colleagues or supervisors), with emphasis on their compounding effects (42); Finally, developing adaptive monitoring mechanisms to track emerging WPV patterns under evolving healthcare challenges, providing data-driven insights for fostering safe and sustainable clinical environments (43).

The findings of this study demonstrate a notably high incidence of WPV among nurses in Lanzhou City, highlighting a prevalent and pressing occupational health issue. WPV significantly undermines nurses' professional wellbeing, inflicting substantial damage on their physical and mental health while adversely affecting their perceptions of the work environment. To address this challenge, healthcare institutions and administrative departments should implement targeted, tiered interventions focusing on high-risk settings and vulnerable populations. Key measures include optimizing clinical workflows to mitigate risk triggers, establishing institutional zero-tolerance policies with transparent reporting mechanisms, delivering competency-based training in de-escalation and crisis communication, and cultivating psychological resilience through structured capacity-building programs. By systematically integrating these strategies, healthcare organizations can effectively reduce WPV incidence, safeguard nurses' holistic wellbeing, and ultimately foster safer practice environments conducive to high-quality patient care delivery.

## Data Availability

The original contributions presented in the study are included in the article/supplementary material, further inquiries can be directed to the corresponding authors.
